# Anti-Oxidative Stress Activity Is Essential for *Amanita caesarea* Mediated Neuroprotection on Glutamate-Induced Apoptotic HT22 Cells and an Alzheimer’s Disease Mouse Model

**DOI:** 10.3390/ijms18081623

**Published:** 2017-07-27

**Authors:** Zhiping Li, Xia Chen, Wenqian Lu, Shun Zhang, Xin Guan, Zeyu Li, Di Wang

**Affiliations:** 1 Department of Pharmacology, College of Basic Medical Sciences, Jilin University, Changchun 130006, China; zpli15@mails.jlu.edu.cn (Z.L.); jldxgx@126.com (X.G.); uclad@outlook.com (Z.L.); 2 School of Life Sciences, Jilin University, Changchun 130006, China; luwq15@mails.jlu.edu.cn (W.L.); zs1225643908@outlook.com (S.Z.)

**Keywords:** *Amanita caesarea*, Alzheimer’s disease, apoptosis, oxidative stress, cholinergic transmitters

## Abstract

*Amanita caesarea*, an edible mushroom found mainly in Asia and southern Europe, has been reported to show good antioxidative activities. In the present study, the neuroprotective effects of *A. caesarea* aqueous extract (AC) were determined in an l-glutamic acid (l-Glu) induced HT22 cell apoptosis model, and in a d-galactose (d-gal) and AlCl_3_-developed experimental Alzheimer’s disease (AD) mouse model. In 25 mM of l-Glu-damaged HT22 cells, a 3-h pretreatment with AC strongly improved cell viability, reduced the proportion of apoptotic cells, restored mitochondrial function, inhibited the over-production of intracellular reactive oxygen species (ROS) and Ca^2+^, and suppressed the high expression levels of cleaved-caspase-3, calpain 1, apoptosis-inducing factor (AIF) and Bax. Compared with HT22 exposed only to l-Glu cells, AC enhanced the phosphorylation activities of protein kinase B (Akt) and the mammalian target of rapamycin (mTOR), and suppressed the phosphorylation activities of phosphatase and tensin homolog deleted on chromosome ten (PTEN). In the experimental AD mouse, 28-day AC administration at doses of 250, 500, and 1000 mg/kg/day strongly enhanced vertical movements and locomotor activities, increased the endurance time in the rotarod test, and decreased the escape latency time in the Morris water maze test. AC also alleviated the deposition of amyloid beta (Aβ) in the brain and improved the central cholinergic system function, as indicated by an increase acetylcholine (Ach) and choline acetyltransferase (ChAT) concentrations and a reduction in acetylcholine esterase (AchE) levels. Moreover, AC reduced ROS levels and enhanced superoxide dismutase (SOD) levels in the brain of experimental AD mice. Taken together, our data provide experimental evidence that *A. caesarea* may serve as potential food for treating or preventing neurodegenerative diseases.

## 1. Introduction

Alzheimer’s disease (AD), a genetically complex neurodegenerative condition typically associated with ageing, is characterized by a decline in cognitive function and poor prognosis [1]. Although the pathomechanism of AD remains largely unknown, the apoptosis of neurons has been hypothesized as one possible cause [2]. In clinics, neurons loss, deposition of amyloid protein, and formation of neurofibrillary tangles within neurons have been observed in brain regions of AD patients during neuropathological examination [3]. Mitochondria-mediated apoptosis is a pathological feature of neurons during AD development that is accompanied by increased production of reactive oxygen species (ROS) [4]. Oxidative stress in human bodies increases with age and is therefore considered an important causative factor in AD, which also causes remarkable accumulation of amyloid-β peptide (Aβ) [5,6]. 

Moreover, glutamate, the principle neurotransmitter in the adult central nervous system [7], inhibits the synthesis of glutathione, leading to the accumulation of ROS [8]. The neurotoxicity of glutamate in HT22 cells (mouse hippocampal neuronal cells) has been characterized and recognized as a common model for in vitro studies of neurodegenerative disease [9]. The AD mouse model established by intragastric AlCl_3_ administration combined with intraperitoneal injection of d-galactose (d-gal) serves as an ideal in vivo model for AD that can imitate AD-like behavior and pathological alterations [10]. 

Unfortunately, no satisfactory therapeutic agents are in use for AD patients. Herbs and/or fungi constitute a huge and underappreciated source of development in biopharmaceutics due to their beneficial health functions [11]. In our fungal group, a polysaccharide isolated from Sparassis crispa protects against l-glutamic acid (l-Glu) induced apoptosis in PC12 cells via the mitochondrial apoptotic pathway [12]. In l-Glu-induced apoptotic cells and the AlCl_3_- and d-gal-developed AD mouse model, Hericium erinaceus aqueous extract displays therapeutic effects on AD, in relation to not only mitochondria-mediated apoptosis, but also to the modulation of neurotransmitters [13]. *Amanita caesarea*, an edible mushroom, can be found in mixed coniferous and deciduous forests in Asia and southern Europe [14]. To date, most studies have focused on the constituent analysis of *A. caesarea* and its culture [15]. Recently, a polysaccharide separated from *A. caesarea* has been reported to exhibit strong antioxidant activities; thus, it may be a useful naturally occurring antioxidant [15]. The potentially beneficial effects of *A. caesarea* on neurodegenerative diseases, especially AD, have not yet been reported. 

In the present study, l-Glu-induced HT22 apoptotic cells and d-gal- and AlCl_3_-induced experimental AD mice were used to investigate the activities of *A. caesarea* aqueous extracts (AC) on AD. Encouragingly, AC protected l-Glu-damaged HT22 cells, as evidenced by improved cell viability, a reduced proportion of apoptotic cells, restored mitochondrial function, regulated apoptosis-related protein expression and protein kinase B (Akt)/mammalian target of rapamycin (mTOR) signal pathway. Furthermore, AC improved behavioral, physiological, and biochemical indexes in experimental AD mice. Our present study suggests that *A. caesarea* may serve as a functional food for the adjuvant therapy of AD.

## 2. Results

### 2.1. AC Ameliorated l-Glu-Induced Cytotoxicity and Apoptosis in HT22 Cells

**Figure 1:**
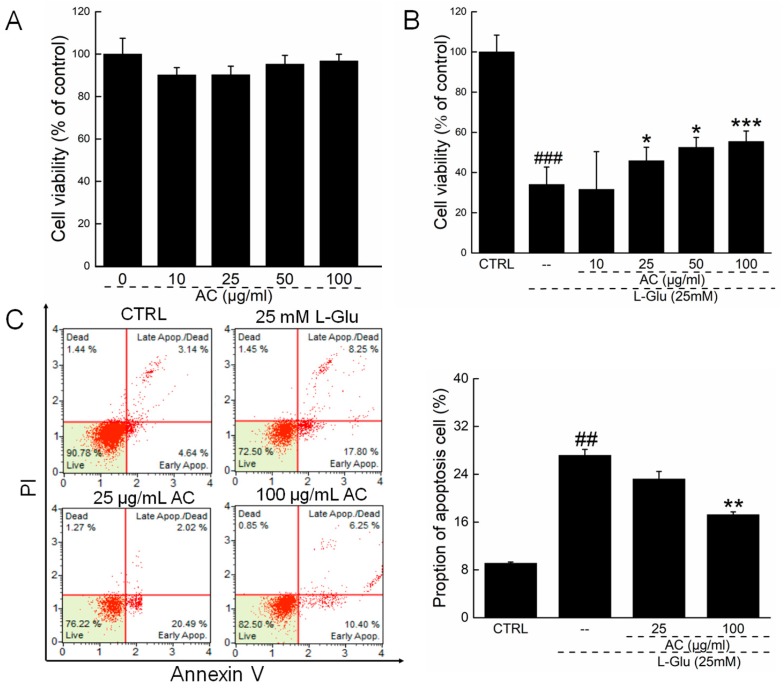
AC ameliorated l-Glu-induced cytotoxicity and apoptosis in HT22 cells. (**A**) AC has no significant influence on HT22 cell viability; (**B**) AC enhanced cell viability in l-Glu-damaged HT22 cells after 24 h co-incubation; (**C**) AC reduced proportion of the apoptotic cells in l-Glu-exposed HT22 cells detected by Annexin V-FITC/PI staining. Data are expressed as mean ± S.D. (*n* = 6). ^##^ *p* < 0.01 and ^###^ *p* < 0.001 vs. CTRL. * *p* < 0.05, ** *p* < 0.01 and *** *p* < 0.001 vs. l-Glu-treated cells.

To detect the influence of AC on HT22 cell, MTT assay was used. HT22 exposed only to AC shows no obvious changes in cell viability (Figure 1A). Reductions in cell viability of over 60% were noted in 24-h l-Glu-exposed HT22 cells (*p* < 0.001; Figure 1B). A 3-h pretreatment with AC at doses of 25, 50, and 100 µg/mL and co-incubation with l-Glu (25 mM) for 24 h enhanced cell viability by 9.4%, 18.2%, and 21.3%, respectively, in HT22 cells compared with the l-Glu group (*p* < 0.05; Figure 1B). Compared with l-Glu-treated cells, 100 µg/mL of AC pretreatment reduced the proportion of apoptotic cells around 10% after a 3h-pretreatment and 24-h co-incubation (27.2 ± 0.76% vs. 17.1 ± 0.54%; *p* < 0.01; Figure 1C).

### 2.2. AC Ameliorated l-Glu-Caused Mitochondrial Dysfunction in HT22 Cells

**Figure 2:**
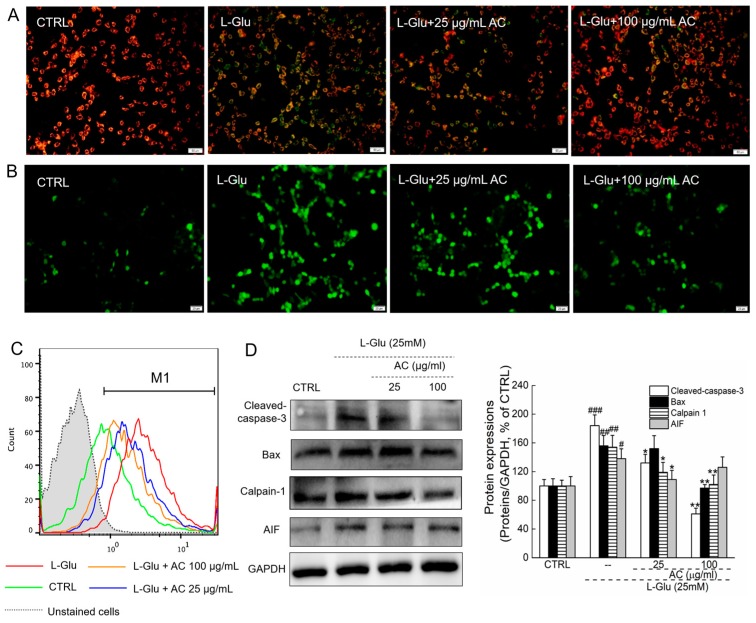
AC ameliorated MMP loss, intracellular ROS and Ca^2+^ over-production, and the apoptotic alternations on the expression levels of proteins. (**A**) AC pretreatment restored the disruption of MMP caused by 12-h l-Glu exposure analyzing by 5,5′,6,6′-tetrachloro-1,1′,3,3′-tetraethyl-imidacarbocyanine iodide staining (JC-1) (*n* = 6) (20×; Scale bar: 25 μm); (**B**) AC pretreatment inhibited the over-accumulation of intracellular ROS caused by 12-h l-Glu exposure detecting by DCFH–DA staining (*n* = 6) (20×; Scale bar: 25 μm); (**C**) AC ameliorated the over-load of intracellular Ca^2+^ caused by 12-h l-Glu exposure analyzing by Fluo 4-AM staining (*n* = 6); (**D**) AC reduced the expression levels of cleaved caspase-3, Bax, calpain 1 and apoptosis-inducing factor (AIF) in l-Glu-exposed HT22 cells after 24-h co-incubation. Quantification data was normalized by GAPDH, expressed as percentage of CTRL and mean ± S.D. (*n* = 6). ^#^ *p* < 0.05 and ^##^ *p* < 0.01 vs. CTRL, * *p* < 0.05 and ** *p* < 0.01 vs. l-Glu-treated cells.

An imbalance in mitochondrial membrane potential (MMP) characterizes the early stage of mitochondrial injury [16]. Intense red fluorescence was noted in untreated cells, indicating a healthy state (Figure 2A). In contrast, 12-h l-Glu exposure significantly decreased MMP as evidenced by the appearance of green fluorescence, which was restored by 3-h AC pretreatment and 12-h co-treatment at doses of 25 and 100 µg/mL (Figure 2A). Overproduction of ROS resulting in oxidative stress can be an important mediator of damage to cell structures [5]. l-Glu exposure for 12 h strongly enhanced the intracellular ROS level, which was inhibited by a 3-h AC pretreatment and 12-h co-treatment at doses of 25 and 100 µg/mL, as indicated by the reduced intensity in the green fluorescence (Figure 2B).

**Figure A1:**
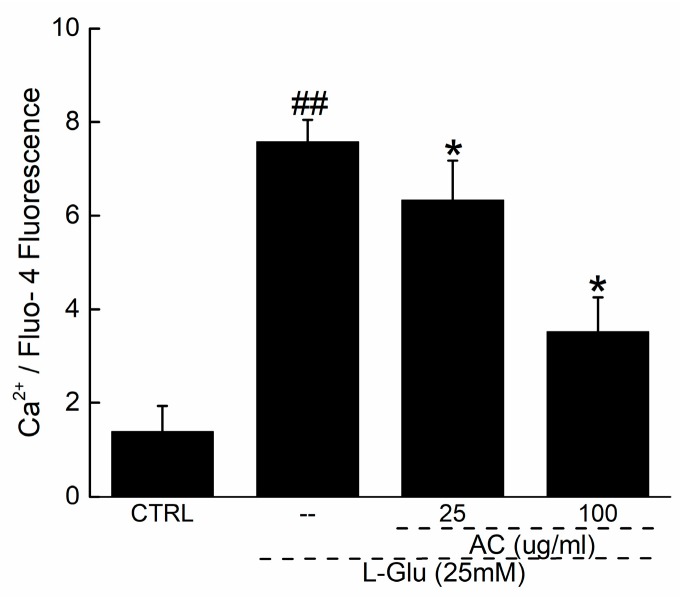
Effect of AC on the over-load of intracellular Ca^2+^ caused by 12-h l-Glu. Quantitative analysis of Ca^2+^ values of fluorescence intensity obtained from Figure 2C (*n* = 6). ^##^ *p* < 0.01 vs. CTRL, * *p* < 0.05 vs. l-Glu-treated cells.

Ca^2+^ overload is a key mediator of l-Glu-induced toxicity, which is associated with mitochondrial function [17]. Compared with control cells (green peak), a 12-h l-Glu exposure caused Ca^2+^ overload (red peak) (*p* < 0.01; Figure A1 and Figure 2C). A 3-h AC pretreatment at doses of 25 and 100 µg/mL (orange and blue peak, respectively) successfully inhibited the high Ca^2+^ levels in HT22 cells (*p* < 0.05; Figure A1 and Figure 2C).

Calpains and AIF are proteins that relate to mitochondrial function [18,19]. l-Glu enhanced the expression levels of calpains, AIF, Bax and cleaved caspase-3 in HT22 cells (*p* < 0.05), whereas AC strongly suppressed these enhancements after 24-h co-incubation (*p* < 0.05; Figure 2D). 

### 2.3. AC Ameliorated l-Glu-Induced Cell Injury by Akt/mTOR Pathway

**Figure 3:**
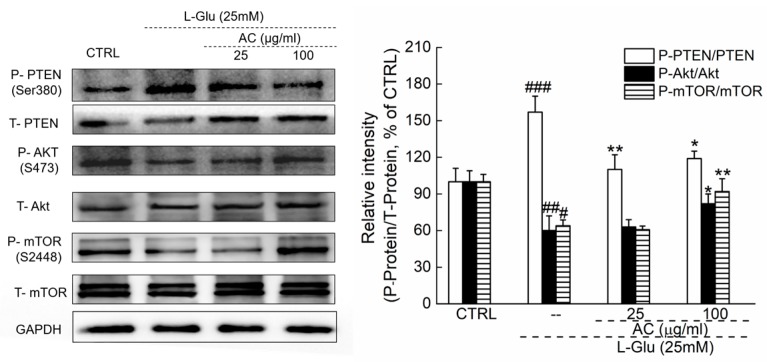
3-h AC pre-exposure regulated the phosphorylation activities of PTEN, Akt and mTOR in l-Glu-exposed HT22 cells. Quantification data of the expressions of P-PTEN, P-Akt and P-mTOR were normalized by corresponding T-pTEN, T-Akt and T-mTOR. Data are expressed as mean ± SD (*n* = 6) and analyzed using one-way ANOVA. ^#^ *p* <0.05, ^##^ *p* < 0.01 and ^###^ *p* < 0.001 vs. CTRL, * *p* < 0.05 and ** *p* < 0.01 vs. l-Glu-treated cells.

The pro-survival and anti-apoptotic Akt signaling is involved in neuronal protection [20]. In l-Glu-exposed HT22 cells, reduced expression levels of P-Akt and P-mTOR and enhanced phosphorylation activities of phosphorylation-tensin homolog deleted on chromosome ten (PTEN) were noted (*p* < 0.05; Figure 3). All these abnormal changes were regulated by 3-h AC pretreatment followed by 24-h co-imbibition (*p* < 0.05; Figure 3).

### 2.4. AC Improved AD-Like Behaviors in d-gal and AlCl_3_ Induced AD Mice

**Figure 4:**
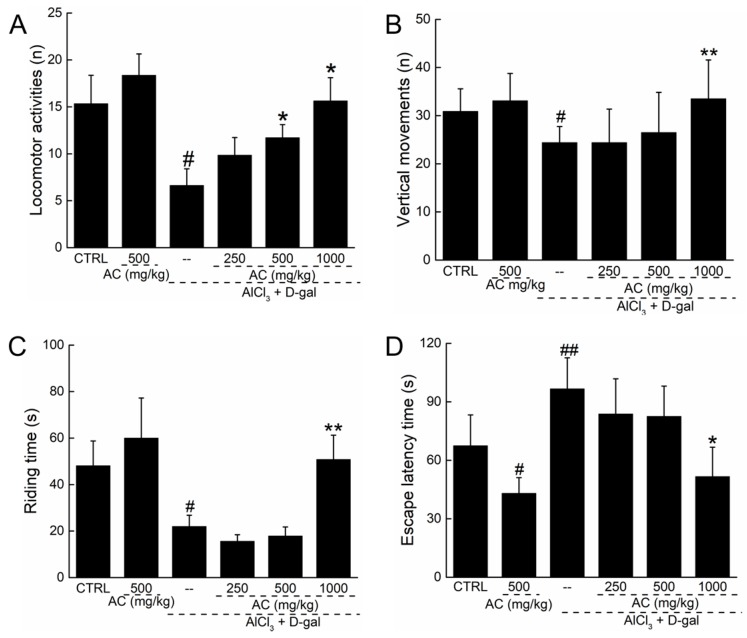
AC improves AD-like behaviors in d-gal and AlCl_3_ development AD mice. Compared with non-treated AD mice, 28-day AC administration enhanced horizontal movements(**A**) and vertical movements (**B**) in locomotor activity test; (**C**) enhanced the remaining time in rotating test; (**D**) reduced the escape latency time in WMT. Data expressed as mean ± S.E.M. (*n* = 12). ^#^ *p* < 0.05 and ^##^ *p* < 0.01 vs. control mice, * *p* < 0.05 and ** *p* < 0.01 vs. AD mice.

In the experimental AD mouse model induced by AlCl_3_ and d-gal, the number of locomotor activities (*p* < 0.05; Figure 4A) and vertical movements (*p* < 0.05; Figure 4B) strongly decreased, and these effects were enhanced by a 28-day AC administration (*p* < 0.05; Figure 4A,B). Compared with normal mice, a reduction of over 68.7% in the remaining time in the rotating test was noted in AD-like mice; in contrast, AC enhanced the remaining time by 12.3% compared with untreated AD mice (*p* < 0.05; Figure 4C). In normal mice, AC treatment alone had no significant effect on mouse movements or on the remaining time in the rotating test (Figure 4A–C). To further explore the protective effects of AC, the Morris water maze test was used to detect its influence on learning and memory in AD-like mice. Compared with other AD-like mice, AC reduced the escape latency by up to 47.1% (51.6 ± 15.1 s vs. 96.6 ± 15.9 s; *p* < 0.05; Figure 4D). AC alone also reduced the escape latency in normal mice (*p* < 0.05; Figure 4D). All data suggest that AC significantly improves memory and physical performance in the AD experimental mouse model.

### 2.5. AC Regulated the Levels of Acetylcholine (Ach), Acetyltransferase (ChAT), and Acetylcholine Esterase (AchE) in the Serum and Brain of AD Mice

The neurotransmitter acetylcholine is a major modulator of learning and memory [21]. AC treatment alone showed no effect on the levels of Ach, ChAT, and AchE in the serum and brain compared with normal mice (Table 1). In AD experimental mice, a significant reduction in Ach and ChAT levels (*p* < 0.05; Table 1) and a significant increase in AchE levels (*p* < 0.05; Table 1) were noted in the serum and brain of AD-like mice. A 28-day AC treatment, especially at 1000 mg/kg, successfully restored all the altered levels of Ach, ChAT, and AchE in the serum and brain of AD mice (*p* < 0.05; Table 1). AC therefore has the capability to reduce cholinergic dysfunction in an experimental mouse model of AD, thereby reducing behavioral dysfunctions.

### 2.6. AC Modulated the Levels of Superoxide Dismutase (SOD) and ROS in the Brains of AD Mice

**Figure 5:**
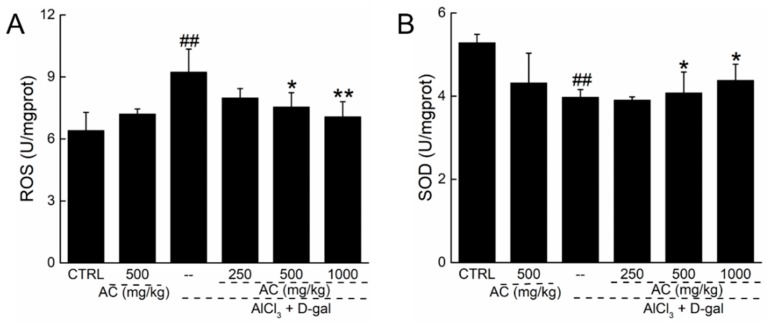
AC ameliorated oxidation stress in brain of AD mice. 28-day AC administration reduced the levels of ROS (**A**); and enhanced the levels of SOD (**B**) in brain of AD-like mice. Data expressed as mean ± S.E.M. (*n* = 12). ^##^ *p* < 0.01 vs. control mice, * *p* < 0.05 and ** *p* < 0.01 vs. AD mice.

Oxidative stress is responsible for cell loss and other pathologies during the development of neurodegenerative diseases [22]. AC alone showed no significant effects on the levels of SOD and ROS in brain (Figure 5). In AD experimental mice, however, a 24.6% reduction in SOD and a 44.7% increase in ROS in the brain were noted (*p* < 0.01; Figure 5), but a 28-day AC administration enhanced SOD levels (*p* < 0.05; Figure 5A) and reduced ROS levels (*p* < 0.05; Figure 5B) in the brain. The antioxidative properties of AC contribute to its neuroprotective effects.

### 2.7. The Effect of AC on the Levels of Aβ 1-42 in Serum and Brain of AD Mice

**Figure 6:**
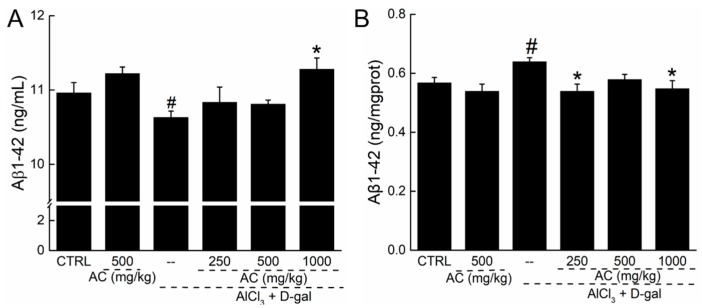
The effect of AC on the levels of Aβ1-42 in serum and brain of AD-like mice. 28-day AC administration enhanced the levels of Aβ1-42 in serum (**A**); but reduced the levels of Aβ1-42 in brain (**B**) of AD-like mice. Data expressed as mean ± S.E.M. (*n* = 12). ^#^ *p* < 0.05 vs. control mice, * *p* < 0.05 vs. AD mice.

The accumulation of Aβ containing neuritic plaques in the brain is a neuropathological feature of AD [23]. AC treatment alone displayed no effects on the levels of Aβ1-42 in the serum and brain of normal mice (Figure 6). However, low levels of Aβ1-42 in serum and high levels of Aβ1-42 in the brain were noted in AD mice (Figure 6). A 28-day administration of AC enhanced the Aβ1-42 levels in serum by 15% (*p* < 0.05; Figure 6A), and reduced levels of Aβ1-42 in the brain by 14.3% (*p* < 0.05; Figure 6B).

## 3. Discussion

AD affects more than 40 million people worldwide, and is predicted to increase exponentially in the coming decade [24]. In the present study, we successfully confirmed the protective effects of AC on AD, as suggested by the improvement in cell viability, reduction in the proportion of apoptotic cells, restoration of MMP dissipation, inhibition of ROS and Ca^2+^ overproduction, regulation of the expression of apoptosis-related proteins, influence of Akt/mTOR signal pathway in l-Glu-damaged HT22 cells, and the enhancement of learning and memory abilities in experimental AD mice associated with the regulation of oxidative stress and the cholinergic system. 

Glutamate inhibits cysteine uptake, results in the downregulation of glutathione, and ultimately causes oxidative stress and necrotic and apoptotic cell processes. AC has been reported to show anti-oxidative effects in cells [25], which led us to further investigate its neuroprotection related to oxidative stress. Mitochondria, the centers of intracellular energy metabolism, can be affected by intracellular Ca^2+^ overload [26]. As Ca^2+^-activated non-lysosomal cysteine proteases [18], the deregulated calpain 1 activity following with the loss of Ca^2+^ homeostasis results in mitochondrial depolarization, cleavage of Bid and AIF [27], and ROS released from the mitochondria into the cytoplasm [18,28]. In a feedback loop, the overaccumulation of ROS in cytoplasm may further cause MMP dissipation [29]. In our present study, we found that AC not only improved MMP dissipation by reducing the raised levels of intracellular ROS and Ca^2+^, but also inhibited the high expression of cleaved caspase-3, calpain 1, AIF, and Bax in l-Glu-exposed HT22 cells. As one of the Bcl-2 family, Bax serves as a hallmark for mitochondrial function detection [30]. The dissipation of MMP triggers caspase-3, which is activated in the cytosol and finally executes apoptosis [31]. However, the pro-survival and anti-apoptotic Akt/mTOR signaling pathway is involved in neuronal protection through the stimulation of neuroprotective factors [32]. Akt deactivation characterizes both caspase-dependent and -independent cell death, and improves mitochondrial function by regulating the expression of the Bcl-2 family [33,34]. As reported, the accumulation of ROS activates multiple pathways including Akt signaling [35]. Activated Akt phosphorylates the downstream mTOR, which regulates cell metabolism by promoting mitochondrial biogenesis and the synthesis of proteins, lipids, and nucleotides [36]. Moreover, as a key negative regulator of Akt/mTOR signaling [37], the phosphorylation activity of PTEN was strongly reduced by 3-h AC pretreatment. Combining all of the data, the neuroprotective effects of AC on l-Glu damaged HT22 cells may be related to improvement in mitochondrial functioning via the modulation of Akt/mTOR signaling. 

While testing the sub-acute toxicity of several carbohydrates, scientists in China first reported that injections of d-gal could induce neurological impairment in rodents [38]. Aluminum itself is neurotoxic in animals, and capable of forming a complex with Aβ that has more ready access to the brain, causing cerebrovascular dysregulation [39].Chronic administration of d-gal and AlCl_3_ leads to deterioration in cognitive and motor skills and AD-like behavior due to brain damage through various mechanisms, including oxidative stress [40,41], dysfunction of the cholinergic system [42,43], biochemical and histopathological alterations, and aggregation of Aβ [39]. By improving AD-like behavior in a d-gal- and AlCl_3_-developed AD-like mice model, AC has confirmed its potential neuroprotective properties in AD-like mice. In line with other studies, we observed elevated ROS that led to a dramatic decrease in SOD in the brain of AD mice, which was significantly improved after AC administration. An imbalance between ROS generation and termination accelerates AD progression [44,45]. Endogenous antioxidants such as SOD are suggested to be a first-line defense mechanism involving oxidative stress [46]. Additionally, AC elevated serum levels of Aβ and decreased the expression of Aβ in the brain. Aβ is normally cleared from the brain into the periphery, and changes in its production or clearance can result in its accumulation in the brain, accompanied by reduced peripheral levels and the clinicopathological manifestations of AD [47]. As a hallmark of AD, Aβ is responsible for spatial memory deficit and cognitive dysfunction [48], which is regulated by oxidative stress through enhancement of the amyloidogenic fragment of APP [46,49]. The data are consistent with the results obtained in HT22 cells, where the antioxidative activities of AC resulted in reducing the learning and memory deficits in AD-like mice. 

In addition to oxidative stress, the impaired cholinergic functionality observed in AD is also responsible for the cognitive decline and memory loss [46]. Ach, a major modulator of learning and memory, is dynamically controlled by the synthesizing enzyme ChAT and the terminating enzyme AchE [50]. The reduced levels of Ach and ChAT were found in the brain of AD patients [51]. In our group, *H. erinaceus* has been confirmed to show protection against AD via dose-dependent enhancement of Ach and ChAT concentrations in both serum and the hypothalamus [13]. In the present study, AC not only enhanced the levels of Ach and ChAT, but also reduced the levels of AchE in the brains of AD-like mice, indicating that the cholinergic function has an important role in protecting against AD. However, further investigation is needed to provide more detailed information related to cholinergic activities during AD development and AC therapy.

In conclusion, use of the l-Glu-induced HT22 apoptotic cell model and the AlCl_3_- and d-gal-developed experimental AD mice model has successfully confirmed the potential neuroprotective properties of AC against AD, which may be related to its modulation of oxidative stress and neurotransmitters levels, especially the cholinergic system.

## 4. Materials and Methods

### 4.1. Preparation of A. caesarea Water Extract (AC)

*A. caesarea* sporocarp (obtained from Yunnan province, China) was extracted in hot water at 80 °C for 3 h twice. In detail, 100 g *A. caesarea* was mashed, soaked in 3 L double distilled (D.D.)-water, heat up to 80 °C for 3 h, then the mixture was filtered and 3 L D.D.-water was added again. The supernatant fluid was collected, concentrated to 280 mL (360 mg/mL), and then freeze-dried by a Freeze dryer (GENESIS, SQ25ES, VirTis, Gardiner, NY, USA). Then the powder was diluted to the concentration for the further experiments.

### 4.2. Cell Culture

HT22 cell, a mouse hippocampal neuron cell line (BNCC, 337709), was cultured in Dulbecco’s Modified Eagle Medium (DMEM) supplemented with 10% fetal bovine serum (FBS), 100 units/mL penicillin and 100 μg/mL of streptomycin at 37 °C in an atmosphere containing 5% CO_2_ and 95% air. All the cell culture materials were purchased from BRL (Grand Island, NY, USA).

### 4.3. Cell Viability Assay

HT22 cells were seeded into the 96-well plates at 5 × 10^3^ cells/well and pre-treated with AC from 0 to 100 μg/mL for 3 h, and then 25 mM of l-Glu (Sigma-Aldrich, St. Louis, MO, USA) were added and co-cultured with AC for another 24 h. AC was diluted with basic medium to final concentration. The final concentration of serum was kept at 1% in both 96-well plates and 6-well plates. 10 μL of 3-(4,5-dimethylthiazol-2-yl)-2,5-diphenyltetrazolium bromide (MTT) at the final concentration of 0.5 mg/mL was added and reacted with the living cells. After the 4-h incubation, the supernatant was removed, and 200 μL of DMSO were added. The optical density at wavelength of 570 nm was read by a Synergy^TM4^ Microplate Reader (BioTek Instruments, Winooski, VT, USA).

### 4.4. Cell Apoptosis Assay

HT22 cells were pretreated with AC at doses of 25 and 100 µg/mL for 3 h, and then co-incubated with 25 mM of l-Glu for another 24 h. Then cells were digested with trypsin, and washed with phosphate-buffer saline (PBS) twice. Then cells were resuspended with PBS and incubated with propidium iodide (PI) and Annexin V for 20 min at room temperature in darkness. The intensity of fluorescence was measured utilizing Muse^TM^ Cell Analyzer from Millipore (Billerica, MA, USA) following manufacturer’s instructions. 

### 4.5. Mitochondrial Transmembrane Potential (MMP) Measurement

JC-1 (Calbiochem, San Diego, CA, USA) staining was used to detect the changes in MMP. Dissolve the dye with basic medium, and warm it up in a water bath at 37 °C with light exposure. HT22 cells were pre-incubated with 25 and 100 μg/mL of AC for 3 h, and then co-incubated with 25 mM of l-Glu for another 12 h. The cells were washed with warmed PBS twice then incubated with dyes. After 15-min staining of 2 μmol/L of JC-1 in darkness at 37 °C, cells were washed with PBS for three times. The red and green fluorescence was recorded with fluorescence microscope (20×; CCD camera, TE2000, Nikon, Tokyo, Japan). The experiment was repeated for six times.

### 4.6. Intracellular ROS Measurement

HT22 cells were pre-incubated with AC (25 and100 μg/mL) for 3 h, and followed by a 12-h co-culture with 25 mM of l-Glu. Fluorescent probe dye was diluted with basic medium and warmed up in a water bath at 37 °C Cells were stained with 10 μM of 2′-7′-dichlorodihydrofluorescein diacetate (DCFH-DA, Sigma-Aldrich, St. Louis, MO, USA) for 15 min in darkness at 37 °C, and then washed with PBS for three times. The green fluorescence, which reflects the intracellular ROS level, was recorded by a Nikon Eclipse TE 2000-S fluorescence microscope (Nikon, Japan). 

### 4.7. Intracellular Ca^2+^ Measurement

Fluo-4-AM (Molecular Probes, Eugene, OR, USA) dye was diluted with DMSO to the concentration of 2 mM, HT22 cells were seeded into 6-well plates at the density of 2 × 10^5^ cells/well, and pre-treated with AC at 25 and100 μg/mL for 3 h, followed by 12-h co-culture with 25 mM of l-Glu. Cells were digested with trypsin and centrifuge for 3 min at 1200 r. Collected cells were resuspended with Hank’s Balanced Salt Solution (HBBS) containing 2 μM Fluo-4-AM. After 15-min staining, cells were washed with HBSS for three times, then subjected to flowcytometric analysis using the Muse™ Cell Analyzer (Millipore, Billerica, MA, USA).

### 4.8. Western Blot

HT22 cells were pre-treated with AC at 25 and 100 μg/mL for 3 h followed by 24-h co-incubation with 25 mM of l-Glu. Then cells were harvested and lysed with RIPA buffer (Sigma-Aldrich, St. Louis, MO, USA) containing 1% protease inhibitor cocktail (Sigma-Aldrich, St. Louis, MO, USA) and 2% Phenylmethanesulfonyl fluoride (PMSF) (Sigma-Aldrich, St. Louis, MO, USA). BCA protein assay kit was used to determine the concentration of the lysed cell protein. 40 µg of protein lysates were separated by 10–12% SDS-PAGE and transferred onto polyvinylidene difluoride (PVDF) membranes (0.45 μm, Merck Millipore, Billerica, MA, USA). Then the membranes were blocked by 5% bovine serum albumin (BSA) for 2 h at room temperature, followed by 12-h incubation of primary antibodies at 4 °C: phosphor (P)-mTOR (S2448) (ab109268), total (T)-mTOR (ab83495), P-Akt (S473) (ab18206), T-Akt (ab106693), cleaved-caspase 3 (ab2302), Bax (ab32503), and Glyceraldehyde-3-phosphate dehydrogenase (GAPDH; ab181602) (Abcam, Cambridge, MA, USA) at 4 °C overnight at, calpain 1 (bs-1099R), AIF (bs-0037R), phospho-PTEN (Ser380) (bs-3350R), PTEN (bs-0686r) (Bioss Inc., Beijing, China). Horseradish peroxidase (HRP)-conjugated secondary antibody was used to bind the primary antibody at 4 °C for 4 h. The protein bands were visualized using an ECL kit (Merck Millipore, Billerica, MA, USA), and the membranes were scanned utilizing an imaging system (BioSpectrum600). The pixel density was quantified by Image J software (National Institutes of Health, Bethesda, MD, USA). 

### 4.9. The Establishment of AD Mouse Model and Drug Treatment Process

The study was approved by the Institution Animal Ethics Committee of Jilin University, and all experiments were carried out in accordance with the Institutional guidelines on the care and use of experimental animals. BALB/c mice (6–8 weeks; 18–20 g) purchased from Norman Bethune University of Medical Science, Jilin University, Changchun, Jilin, China (SCXK(JI)-2011-0003) were maintained on a standard 12-h:12-h light/dark cycle at 23 ± 1 °C. Food and water were fed autoclaved standard chow and ad libitum.

**Figure 7:**
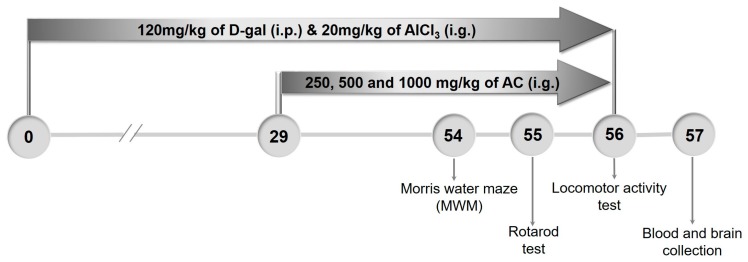
Schematic of experiments. The AD mouse model was established by given AlCl_3_ (20 mg/kg, i.g.) and d-gal (120 mg/kg i.p.) daily for 56 days. From the 29th day, the mice were given AC at doses of 250,500 and 1000 mg/kg daily for 28 days. Behavioral tests were performed starting from the 54th day.

The process of establishing the AD mouse model and the drug administration was shown in Figure 7. Generally, mice were randomly divided into 6 groups (*n* = 20/group). The model mice and AC-treated mice were intraperitoneally (i.p.) injected with 120 mg/kg of d-gal (Sigma-Aldrich, St. Louis, MO, USA) and intragastrically (i.g.) administrated with 20 mg/kg of AlCl_3_ (Sigma-Aldrich, St. Louis, MO, USA) once a day for 56 days. From the 29th day, AC-treated mice were intragastrically treated with 250, 500 and 1000 mg/kg of AC everyday; meanwhile, model mice were intragastrically treated with D.D. water. Control mice were intraperitoneally and intragastrically treated with saline throughout the whole experiment. AC single treated mice were intraperitoneally and intragastrically treated with saline throughout the whole experiment and intragastrically treated with 500 mg/kg of AC from the 29th day.

### 4.10. Behavioral Tests

Locomotor activity test. Each mouse was separately placed into a dark square of the multichannel activity box for testing the independent activity of animals (ZZ-6, Chengdu Techman Software Co., Ltd., Chengdu, China) to observe their reaction in the darkness. Mice were given two minutes to adapt the environment before the formal test. The horizontal and vertical activities within 5 min were recorded.

Rotarod performance test. The rotating rod test reflects the neurological deficits of mice. Mice were pre-trained for 1 min for three times before the formal experiment. For the analysis, mice were placed on the rotating cylinder (ZB-200, Chengdu Techman Software Co., Ltd., Chengdu, China) with the speed of 20 rpm, and the time when mice fell off was recorded. 

Morris water maze test. The Morris water maze test (MWM) was used to evaluate the learning and memory abilities of mice. The maze was a circular pool filled with 10 cm depth water (24–26 °C) containing 1 L of milk. Mice were trained quietly with the same position of operator for five days before the formal test. The latency from immersion of mice into the pool to escape onto the hidden platform was recorded. On the test day, mice were subjected to a 120 s probe trial in which the platform was obscure from the pool. The time spent within 120 s probe test time in target quadrant was recorded. 

### 4.11. Biochemical Analysis

After behavioral tests, blood was sampled from caudal vein of each mouse, and then, mice were anesthetized and decapitated quickly. Brains of mice were removed immediately and placed onto ice. The brain was homogenized in ice-cold PBS (*w*/*v*: 1–5) and the protein concentration was determined using BCA assay kit.

The levels of Ach, AchE, ChAT and Aβ 1-42 in serum and brain were measured by enzyme-linked immunosorbent assay (ELISA) according to the procedures provided by the related assay kits (Nanjing Jiancheng Bioengineering Institute, Nanjing, China). The levels of ROS and SOD in brain were measured by ELISA kits (Nanjing Jiancheng Bioengineering Institute, Nanjing, China).

### 4.12. Statistical Analysis

Data were expressed as mean ± S.D. in cells, and mean ± S.E.M in mouse experiments. SPSS 16.0 software (IBM Corporation, Armonk, NY, USA) was used to analyze the data. The statistical significance was determined by the One-way analysis of variance (ANOVA) followed by post hoc multiple comparisons (Dunn’s test). The value of *p* < 0.05 was considered significant.

## Figures and Tables

**Table 1:** AC regulated the levels of Ach, ChAT and AchE in the serum and brain.

Groups	Neurotransmitters	CTRL	AC (500 mg/kg)	AlCl_3_ + d-gal			
				CTRL	AC (mg/kg)		
					250	500	1000
Serum	Ach (μg/mL)	115.4 ± 5.8	107.2 ± 6.6	87.2 ± 8.4 ^#^	98.3 ± 5.5	89.1 ± 3.1	108.8 ± 4.2 *
	AchE (nmol/ L)	9.7 ± 0.2	9.6 ± 0.1	11.2 ± 0.3 ^#^	10.7 ± 0.1	9.8 ± 0.2 *	9.6 ± 0.1 *
	ChAT (pg/mL)	181.6 ± 8.3	195.7 ± 23.2	163.4 ± 18.2 ^#^	182.7 ± 9.9	204.1 ± 13.7 *	204.1 ± 15.2 *
Tissue	Ach (μg/mgprot)	2.1 ± 0.1	2.4 ± 0.2	1.3 ± 0.1 ^##^	1.3 ± 0.2	1.2 ± 0.1	2.1 ± 0.2 *
	AchE (nmol/gprot)	0.28 ± 0.03	0.21 ± 0.02	0.47 ± 0.06 ^#^	0.48 ± 0.06	0.37 ± 0.02 *	0.37 ± 0.05 *
	ChAT (pg/mgprot)	6.9 ± 0.5	7.2 ± 0.3	5.0 ± 0.1 ^#^	5.1 ± 0.6	7.2 ± 0.3 *	7.3 ± 0.4 *

28-day AC treatment strongly enhanced the levels of Ach and ChAT, and reduced the levels of AchE in serum and brain compared with non-treated AD mice. Data expressed as mean ± S.E.M. (*n* = 12). ^#^ *p* < 0.05 and ^##^ *p* < 0.01 vs. control mice, * *p* < 0.05 vs. AD mice.

## Abbreviations

Ach	Acetylcholine
AchE	Acetylcholine esterase
AC	*A. caesarea* water extracts
AD	Alzheimer’s disease
Akt	Protein kinase B
ANOVA	A one-way analysis of variance
Aβ	Amyloid beta
ChAT	Choline acetyltransferase
d-gal	d-galactose
ELISA	Enzyme-linked immunosorbent assay
GAPDH	Glyceraldehyde-3-phosphate dehydrogenase
l-Glu	l-glutamic acid
MMP	Mitochondrial membrane potential
mTOR	Mammalian target of rapamycin
ROS	Reactive oxygen species
SOD	Superoxide dismutase
